# Supplementary material for: Causal Relationship between Aging and Anorexia Nervosa: A White-Matter-Microstructure-Mediated Mendelian Randomization Analysis
Source: Biomedicines. 2024 Aug 16;12(8):1874. doi: 10.3390/biomedicines12081874 (PMC11351342; doi:10.3390/biomedicines12081874)
Supplement: Supplementary file 1 [file biomedicines-12-01874-s001.zip › Supplementary Figure/Figure S7/Single SNP plot of MR analysis for ubm-b-1958 to AN.pdf]

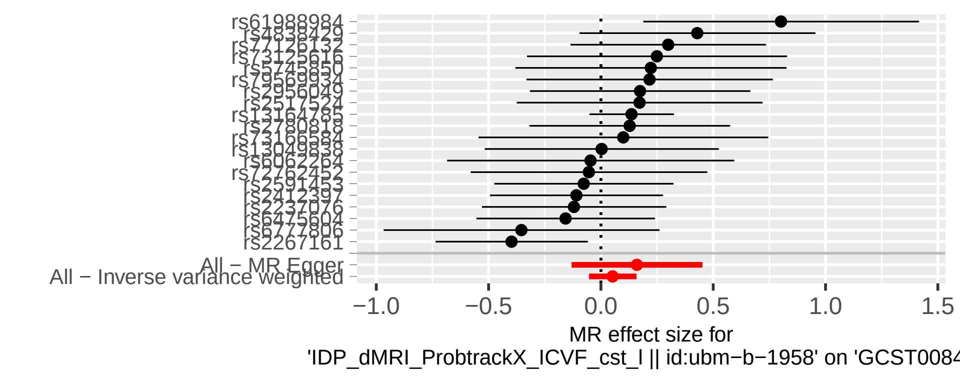

rs61988984
rs77126132
rs73125616
rs79569934
rs2517524
rs13164785
rs73166584
rs72762452
rs2591453
rs2412397
rs2237076
rs6475604
rs2267161
All − MR Egger
All − Inverse variance weighted
−1.0
−0.5
0.0
0.5
1.0
1.5
MR effect size for
'IDP_dMRI_ProbtrackX_ICVF_cst_l || id:ubm−b−1958' on 'GCST0084
